# Expansion of circulating peripheral TIGIT+CD226+ CD4 T cells with enhanced effector functions in dermatomyositis

**DOI:** 10.1186/s13075-020-02397-4

**Published:** 2021-01-07

**Authors:** Wenli Li, Chuiwen Deng, Hanbo Yang, Xin Lu, Shanshan Li, Xia Liu, Fang Chen, Lida Chen, Xiaoming Shu, Lu Zhang, Qingyan Liu, Guochun Wang, Qinglin Peng

**Affiliations:** 1 Department of Rheumatology, China-Japan Friendship Hospital, Ying Hua East Road, Chao Yang District, Beijing, 100029 People’s Republic of China; 2 Department of Rheumatology and Clinical Immunology, Peking Union Medical College Hospital, Chinese Academy of Medical Sciences and Peking Union Medical College, Beijing, People’s Republic of China; 3 Department of Blood Transfusion, China-Japan Friendship Hospital, Beijing, People’s Republic of China

**Keywords:** T cell Ig and ITIM domain, CD226, Dermatomyositis, Co-inhibitory receptor, Co-stimulatory receptor

## Abstract

**Background:**

T cell Ig and ITIM domain (TIGIT)/CD226 pathway has a critical role in regulating T cell responses and has come to the forefront in cancer as a promising immunotherapeutic target. However, its role in autoimmune diseases is just beginning to be elucidated. Dermatomyositis (DM) is an autoimmune disease, in which T cell dysregulation plays a pivotal role, and importantly, it is a common immune-related adverse event in response to treatment of cancers with immune checkpoint inhibitors, but no studies have implicated the TIGIT/CD226 axis in DM.

**Methods:**

We recruited 30 treatment-naïve DM patients and 26 healthy controls. Flow cytometry analysis was used to investigate the co-expression of TIGIT and CD226 on T cells in blood samples. Magnetic bead or FACS-based cell isolation, T cell proliferation assay, and intracellular cytokine staining were performed to analyze the functions of different TIGIT/CD226 phenotypes. Recombinant proteins CD155, CD112, and anti-CD226 antibodies were used to suppress the function of TIGIT/CD226-expressing CD4 T cells.

**Results:**

Four distinct subsets of T cells based on TIGIT/CD226 co-expression, TIGIT+CD226−, TIGIT+CD226+, TIGIT−CD226+, and TIGIT−CD226−, were identified and characterized in DM patients. Our data showed that the function of CD4 T cell subset varied by the TIGIT/CD226 phenotype. An elevated TIGIT+CD226+ CD4 subset with enhanced effector function was observed in patients with DM, especially the patients complicated with interstitial lung disease. This subpopulation was closely related to DM activity and decreased significantly in DM remission after treatment. Furthermore, the effector function of TIGIT+CD226+ CD4 subset could be suppressed by blocking CD226.

**Conclusion:**

Our data revealed that the TIGIT and CD226 expression profiles could be used to identify functionally distinct subsets of CD4 T cells and TIGIT+CD226+ CD4 T cells is a significant subset in DM with enhanced frequency and effector function. This abnormal subset could be suppressed by blocking CD226, providing insight into the therapeutic target of the TIGIT/CD226 axis.

## Background

Dermatomyositis (DM) is an autoimmune inflammatory disease characterized clinically by skin manifestations and muscle weakness and histopathologically by inflammatory infiltrates in the muscle and skin. T cells likely play an important role in DM, as indicated by the predominance of T cells in inflammatory infiltrates from muscle biopsies [1, 2]. Although T cell dysregulation is known to contribute to DM pathogenesis, the mechanism remains largely unclear.

The expression of immune checkpoint receptors has been shown to play a critical role in proper contraction of effector T cell responses [3]. Treating patients with DM with abatacept, a fully human soluble recombinant fusion protein consisting of the co-inhibitory receptor CTLA-4 and Fc domain of human IgG1, reduced disease activity in nearly half of patients monitored. The manual muscle test scores of all patients had improved significantly after treatment [4]. However, an increasing number of studies have suggested that DM develops as an immune-related adverse event in response to treatment of cancers with immune checkpoint inhibitors (e.g., CTLA-4 inhibitor, ipilimumab) [5]. These results suggest that an altered balance between co-stimulatory and co-inhibitory molecules on T cells contributes to DM pathogenesis.

T cell Ig and ITIM domain (TIGIT) is a newly identified co-inhibitory receptor, which, together with CD226, forms a pathway that closely parallels the CD28/CTLA-4 signaling pathway. Analogous to CD28 and CTLA-4, CD226 and TIGIT compete for the same ligands (CD155 and CD112), but these receptor-ligand interactions lead to different outcomes: engagement of CD226 enhances T cell activity, whereas engagement of TIGIT inhibits T cell responses [3, 6]. In cancer, TIGIT is overexpressed and CD226 is underexpressed, and the TIGIT/CD226 pathway has gained attention as a potential clinical target for improving antitumor immune responses [7, 8].

Genome-wide association studies have linked allelic variants in the TIGIT/CD226 pathway to several autoimmune diseases such as type 1 diabetes, rheumatoid arthritis, and multiple sclerosis. However, the role of the TIGIT/CD226 axis in autoimmune diseases remains unclear [9, 10].

Despite the critical roles of this checkpoint axis in immunoregulation, little is known about the expression profile and cellular distribution of T cells expressing TIGIT and CD226. Additionally, no studies have implicated the TIGIT/CD226 axis in the pathogenesis of DM. Therefore, we investigated the expression profiles of these checkpoint molecules on peripheral T cells and their roles in DM disease activity.

## Materials and methods

### Patients

A total of 30 newly diagnosed and untreated patients with DM from China-Japan Friendship Hospital were enrolled in this study from September 2017 to June 2019. The clinical diagnosis of DM was based on the 2017 European League Against Rheumatism/American College of Rheumatology classification criteria [11]. According to the criteria, all the patients were “definite IIM,” with a probability of ≥ 90%, corresponding to a score of ≥ 7.5 (≥ 8.7 with muscle biopsy). The clinical diagnosis of DM was further subclassified using the classification tree of the criteria. Most of the enrolled patients (28/30) had muscle biopsies performed. Twenty-six age- and sex-matched healthy donors without autoimmune disease were also included in the study as healthy controls (HCs). Peripheral blood samples were collected from all participants in heparin sodium anticoagulant and stored at room temperature until use. These samples were obtained under a protocol approved by the Ethics Committee of China-Japan Friendship Hospital, and this study was conducted in accordance with the Declaration of Helsinki guidelines. All patients signed informed consent for participation in this study.

### Clinical characteristics

Clinical manifestations, laboratory measurements, and physical examination details of all patients were recorded at the time of blood draw. The disease duration was defined as the time span since onset of first symptom. ILD was diagnosed using high-resolution computed tomography of the lungs, with at least one of the following abnormalities: parenchymal micronodules and nodules, irregular linear opacities, irregularity of the interfaces between peripheral pleura and aerated lung parenchyma, ground-glass opacities, honeycombing, and traction bronchiectases or bronchiolectases [12]. The diagnosis of dysphagia was based on specific questioning of myositis patients regarding swallowing difficulties, and if necessary, further examinations could be carried out to evaluate esophageal function, including esophageal manometry, barium-swallow examination, or endoscopic examination [13]. Disease activity was evaluated using the 2005 myositis disease activity assessment tool established by the International Myositis Assessment and Clinical Studies Group [14]. The forced vital capacity (% predicted), forced expiratory volume in 1 s (% predicted), and the diffusing capacity of the lungs for carbon monoxide (DLCO) (% predicted) were evaluated to represent pulmonary function. Myositis-specific autoantibodies (MSAs) assayed using line immunoassays (Euroimmun, Luebeck, Germany), which provides a qualitative in vitro evaluation for human autoantibodies of the IgG class to 16 different antigens in serum: Mi-2α, Mi-2β, TIFγ, MDA5, NXP2, SAE1, Ku, PM-Scl100, PM-Scl75, Jo-1, SRP, PL-7, PL-12, EJ, OJ, and Ro-52.

### Flow cytometric analysis for phenotype measurement

Six-color flow cytometry was used to determine the TIGIT and CD226 expression profiles on CD4/CD8 T cells. The following anti-human monoclonal antibodies were used to stain cells: allophycocyanin (APC)-H7-labeled monoclonal anti-CD3, peridin-chlorophyll protein (PerCP)-CY5.5-labeled monoclonal anti-CD4, phycoerythrin (PE)-CY7-labeled monoclonal anti-CD8, APC-labeled monoclonal anti-CD25, and fluorescein isothiocyanate (FITC)-labeled monoclonal anti-CD226, purchased from BD Bioscience (Franklin Lakes, NJ, USA), and PE-labeled monoclonal anti-TIGIT, purchased from Thermo Fisher Scientific (Waltham, MA, USA).

**Fig. 1:**
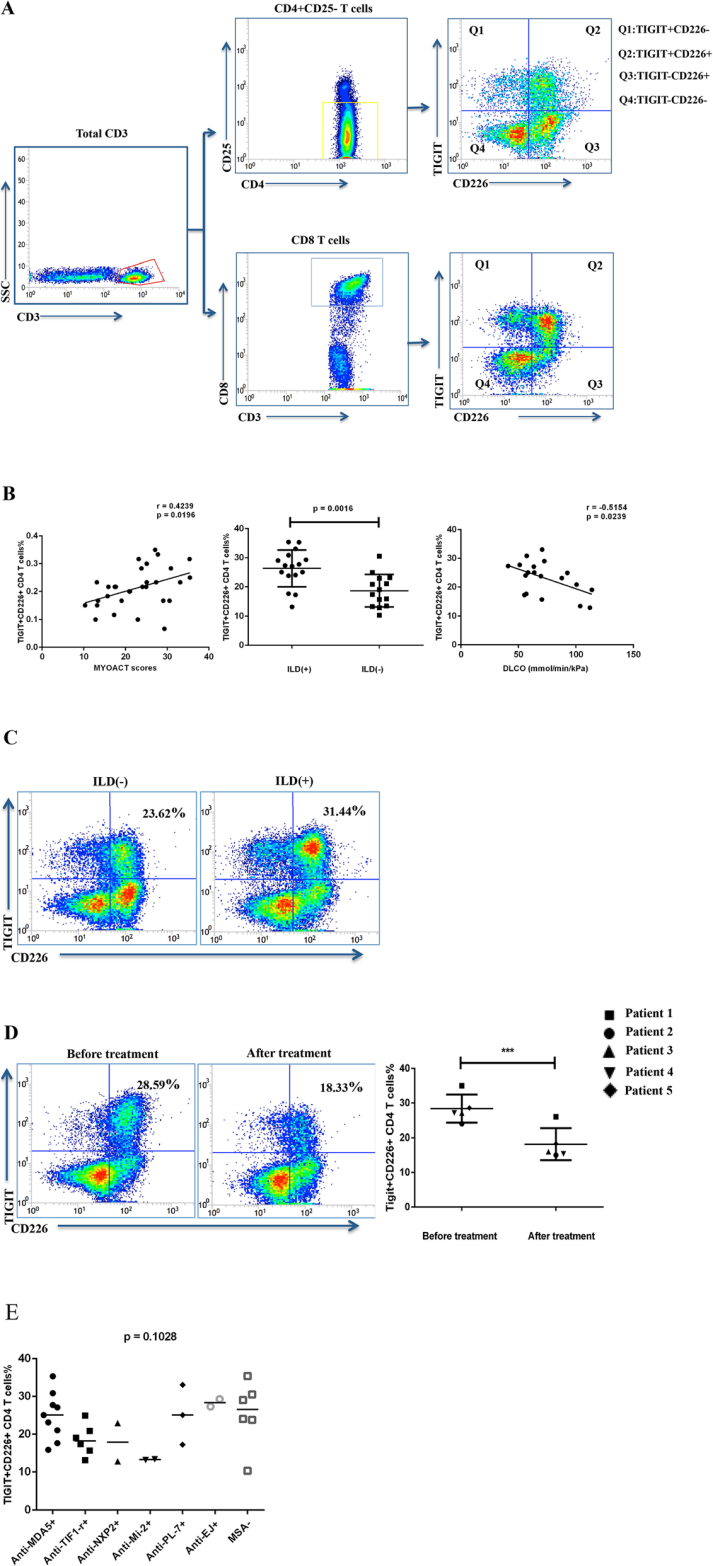
TIGIT+CD226+ CD4 T cell frequencies were highly correlated with DM disease activity. **a** Multi-parameter strategy for assessing the cell surface expression of TIGIT and CD226 on T cells. **b** Frequency of TIGIT+CD226+ CD4 T cell subset was positively associated with disease activity and closely related to ILD involvement. DM patients with ILD (*n* = 16), DM patients without ILD (*n* = 14), and healthy controls (*n* = 26). Spearman’s correlation analysis was used to test for correlation; two groups were compared using unpaired two-tailed *t* test. Data are shown as the mean ± SD. **c** Representative FACS plots showing the percentages of TIGIT+CD226+ T cell/CD4+ T cells in DM patient with ILD and DM patient without ILD. **d** Representative FACS plots and a scatter plot showing decreased percentages of TIGIT+CD226+ CD4 T cells following treatment with medium dose glucocorticoids and disease-modifying anti-rheumatic drugs (*n* = 5). Comparison between two groups was carried out using paired two-tailed *t* test. Data are shown as the mean ± SD. **e** One-way ANOVA test was used to compare the means of TIGIT+CD226+ CD4 T cells levels between MSAs specific subtypes. *p* values < 0.05 were considered significant, *p < 0.05, **p < 0.01, ***p < 0.001

To characterize T cells with different TIGIT/CD226 phenotypes, 100 μL of whole blood cells was incubated with anti-CD3 APC-H7, anti-CD4 PerCP-CY5.5, anti-CD8 PE-CY7, anti-CD25 APC, anti-TIGIT PE, and anti-CD226 FITC as well as with their respective isotype control antibodies according to the manufacturer’s instructions. After 20 min of incubation at room temperature, red blood cells were hemolyzed in 2 mL of 1× fluorescent-activated cell sorting (FACS) lysing solution, and then the leukocytes were centrifuged, washed, and suspended in 500 μL of phosphate-buffered solution for flow cytometry. The samples were processed on a FACS JAZZ instrument. The gating strategies are shown in Fig. 1a, and the results were analyzed using FACS software.

### Cell isolation

Peripheral blood mononuclear cells were isolated from whole blood samples of patients with DM and HCs by Ficoll gradient centrifugation. CD4+CD25− T cells were isolated via magnetic negative selection using a CD4+CD25+ regulatory T cell isolation kit (Miltenyi Biotec, Gladbach Bergisch, Germany), and CD3+CD4+CD25–TIGIT– T cells were sorted on a FACSAria instrument.

### Cell activation and intracellular staining

To analyze the intracellular cytokine production potential of T cells, 1 × 10^6^ purified CD4+CD25– T cells were first stimulated with Leukocyte Activation Cocktail (2 μl/test, BD Biosciences, Franklin Lakes, NJ, USA) for 4 h in a 37 °C humidified CO_2_ incubator. The activated cells were harvested for surface marker staining (PE-labeled monoclonal anti-TIGIT and FITC-labeled monoclonal anti-CD226). After fixation and permeabilization using a Cytofix/Cytoperm kit (BD Biosciences, Franklin Lakes, NJ, USA), the fixed cells were stained with intracellular antibodies PerCP-Cy 5.5-labeled monoclonal anti-interferon γ (IFN-γ) and APC-labeled anti-tumor necrosis factor α (TNF-α) (BD Biosciences, Franklin Lakes, NJ, USA) for 20 min in the dark at room temperature. Each experiment was performed on a FACS JAZZ instrument, and the data were analyzed using FlowJo software (Tree Star, Ashland, OR, USA).

To analyze expression of the late activation marker HLA-DR [15], 1 × 10^6^ purified CD4+CD25- T cells were stained with anti-TIGIT PE, anti-CD226 FITC, and HLA-DR APC (BD Biosciences, Franklin Lakes, NJ, USA) and then analyzed on a FACS JAZZ flow cytometer.

### T cell proliferation assay

For the proliferation assay, 1 × 10^6^ purified CD4+CD25– T cells were labeled with the carboxyfluorescein succinimidyl ester (CFSE) (1 μM; Invitrogen, Carlsbad, CA, USA) in 1 mL pre-warmed phosphate-buffered saline, followed by incubation for 10 min at room temperature and neutralization with pre-chilled complete medium containing 10% fetal bovine serum. CFSE-labeled cells were re-suspended and cultured in complete medium in the presence of plate-bound anti-CD3 (coating concentration was 2.5 μg/mL, Biolegend, San Diego, CA, USA) and soluble anti-CD28 (2.5 μg/mL, Biolegend, San Diego, CA, USA) in 24-well flat-bottom plates for 72 h. After culture, the cells were collected for anti-TIGIT APC and anti-CD226 PerCP-CY5.5 staining and then analyzed to determine the CFSE intensities. Each experiment was performed and analyzed using a FACS JAZZ instrument.

### In vitro recombinant proteins/antibodies treatment

The recombinant proteins CD155 (0.5 μg/mL; Biovision, Milpitas, CA, USA), CD112 (0.5 μg/mL; Sinobiological, China), and anti-CD226 (0.5 μg/mL; BD Pharmingen) were used as drugs to downregulate the activity of different CD4 T cell subsets. The cytokine production potential of different CD4+ T cell subsets was analyzed in the presence of these proteins.

### Statistical analysis

All analyses were performed using SPSS (version 19.0, SPSS, Inc., Chicago, IL, USA) and GraphPad Prism (version 6.0, GraphPad, Inc., La Jolla, CA, USA) software. For continuous variables, the results were expressed as the mean ± SD; comparisons between two groups were carried out using paired or unpaired two-tailed *t* tests. One-way ANOVA test was used for multiple mean comparisons. For nonparametric distribution data, the results were described as the median and range; differences between groups were assessed by Mann-Whitney *U* tests. Spearman’s correlation analysis was used to test for correlation. *p* values less than 0.05 were considered as statistically significant.

## Results

### Clinical characteristics of patients with DM

A total of 30 patients with DM and 26 sex- and age-matched HCs were recruited. Clinical and laboratory parameters of the enrolled subjects are presented in Table 1.

**Table 1:** Clinical and laboratory features of enrolled individuals

	DM	HCs
Number	30	26
Age (years)	45.3 ± 12.5	44.8 ± 10.7
Sex (male to female)	10:16	10/16
Disease duration (months)	3 (1–48)	NA
Major clinical features		
Skin rash (%)	100% (30/30)	NA
Muscular weakness (%)	60% (18/30)	NA
Arthralgia (%)	37% (11/30)	NA
Dysphagia (%)	20% (6/30)	NA
ILD (%)	53% (16/30)	NA
Malignancy (%)	3% (1/30)	NA
MYOACT score (mean ± SD)	0.213 ± 0.072	NA
Major laboratory features		
CK (IU/L), median (IQR)	326 (130–4852)	NA
LDH (IU/L), median (IQR)	262 (40–2953)	NA
MSA positive (%)	80% (24/30)	NA
*Anti-MDA5+* (%)	38% (9/24)	NA
*Anti-TIF1-r+* (%)	25% (6/24)	NA
*Anti-NXP2+* (%)	8% (2/24)	NA
*Anti-Mi-2+* (%)	8% (2/24)	NA
*Anti-PL-7+* (%)	13% (3/24)	NA
*Anti-EJ+* (%)	8% (2/24)	NA
CD4 T cells (cells/μl)	421 (287–1377)	NA
CD8 T cells (cells/μl)	209 (60–681)	NA

*ILD* interstitial lung disease, *ANA* antinuclear antibodies, *CK* creatine kinas, *IQR* interquartile range, *LDH* lactate dehydrogenase, *MSA* myositis-specific antibodies, *NA* not applicable

### TIGIT+CD226+ CD4 T cell frequency was significantly elevated in patients with DM

Based on TIGIT and CD226 expression, we divided the T cells into four subsets: TIGIT+CD226– (Q1), TIGIT+CD226+ (Q2), TIGIT−CD226+ (Q3), and TIGIT−CD226− (Q4). Six-color flow cytometry was performed using the gating strategies shown in Fig. 1a. The distributions of different T cells subsets are shown in Table 2. Compared with HCs, increased percentages of TIGIT+CD226+ CD4 T cells (22.76 ± 7.063% vs. 18.87 ± 5.604%, *p* = 0.0281) and decreased frequencies of TIGIT–CD226+ CD4 cells (20.96 ± 10.15% vs. 29.85 ± 7.492%, *p* = 0.0005) were observed in patients with DM. In contrast, there was no significant difference in the expression profiles of TIGIT/CD226 on CD8 T cells between patients with DM and HCs.

**Table 2:** TIGIT and CD226 expression on T cells of patients with DM and healthy controls

	DM patients (*n* = 30)	Healthy controls (*n* = 26)	*p* value
CD4 T cell (percentages)			
TIGIT+CD226−%	6.995 ± 2.253	6.957 ± 1.871	*p* = 0.9459
TIGIT+CD226+%	22.76 ± 7.063	18.87 ± 5.604	*p* = 0.0281
TIGIT–CD226+%	20.96 ± 10.15	29.85 ± 7.492	*P* = 0.0005
TIGIT–CD226−%	49.29 ± 16.41	43.17 ± 12.78	*p* = 0.1300
CD8 T cell (percentages)			
TIGIT+CD226−%	13.66 ± 7.037	10.52 ± 4.328	*p* = 0.0534
TIGIT+CD226+%	35.26 ± 14.63	30.86 ± 10.45	*p* = 0.2085
TIGIT–CD226+%	19.00 ± 13.61	20.75 ± 8.169	*p* = 0.5709
TIGIT–CD226–%	32.07 ± 14.80	37.87 ± 14.59	*p* = 0.3003

### TIGIT+CD226+ T cell frequency was correlated with DM disease activity

Following evaluation of the TIGIT and CD226 expression profiles on T cells from patients with DM, the association between these profiles and disease activity was explored. The frequency of TIGIT+CD226+ CD4 T cells was positively correlated with the Myositis Disease Activity Assessment (MYOACT) scores (Fig. 1b, *n* = 30, *r* = 0.4239, *p* = 0.0196). We also examined the relationship between TIGIT+CD226+ CD4 T cells and clinical manifestations and observed a significantly higher frequency of this subset in DM patients with interstitial lung disease (ILD) (*n* = 16) than in patients without ILD (*n* = 14) (Fig. 1b, 26.34 ± 6.311% vs. 18.67 ± 5.614%, *p* = 0.0016; representative FACS plots are shown in Fig. 1c). Further, TIGIT+CD226+ CD4 T cell frequencies were negatively correlated with DLCO (Fig. 1b, *n* = 19, *r* = − 0.5154, *p* = 0.0239).

After recruitment, all the patients were treated with corticosteroids at doses of 0.5–1 mg/kg, and 80% of the patients (24/30) were administered one or more immunosuppressants, including methotrexate, cyclophosphamide, cyclosporin A, azathioprine, intravenous immunoglobulin, hydroxychloroquine, or mycophenolate mofetil. Five patients were reexamined after treatment, and all were considered to be in remission with significantly decreased MYOACT global disease scores (0.2375 ± 0.095 vs. 0.1050 ± 0.066, *p* = 0.0081). As expected, significantly decreased percentages of TIGIT+CD226+ CD4 T cells were observed in these patients with DM after treatment compared to those before treatment (28.37% ± 4.064% vs. 18.14 ± 4.578%, *p* < 0.0001). A representative case and scatter plot are shown in Fig. 1d.

Stratification analyses based on MSA-specific subtypes were also performed and the one-way ANOVA test showed that the differences of TIGIT+CD226+ CD4 T cells levels between MSAs specific subtypes were not statistically significant (*p* = 0.1028, Fig. 1e). Because of the limited number of patients included in this study, the analyses did not cover all MSA subtypes.

### CD4 T cell subset function varied by TIGIT/CD226 phenotype

**Fig. 2:**
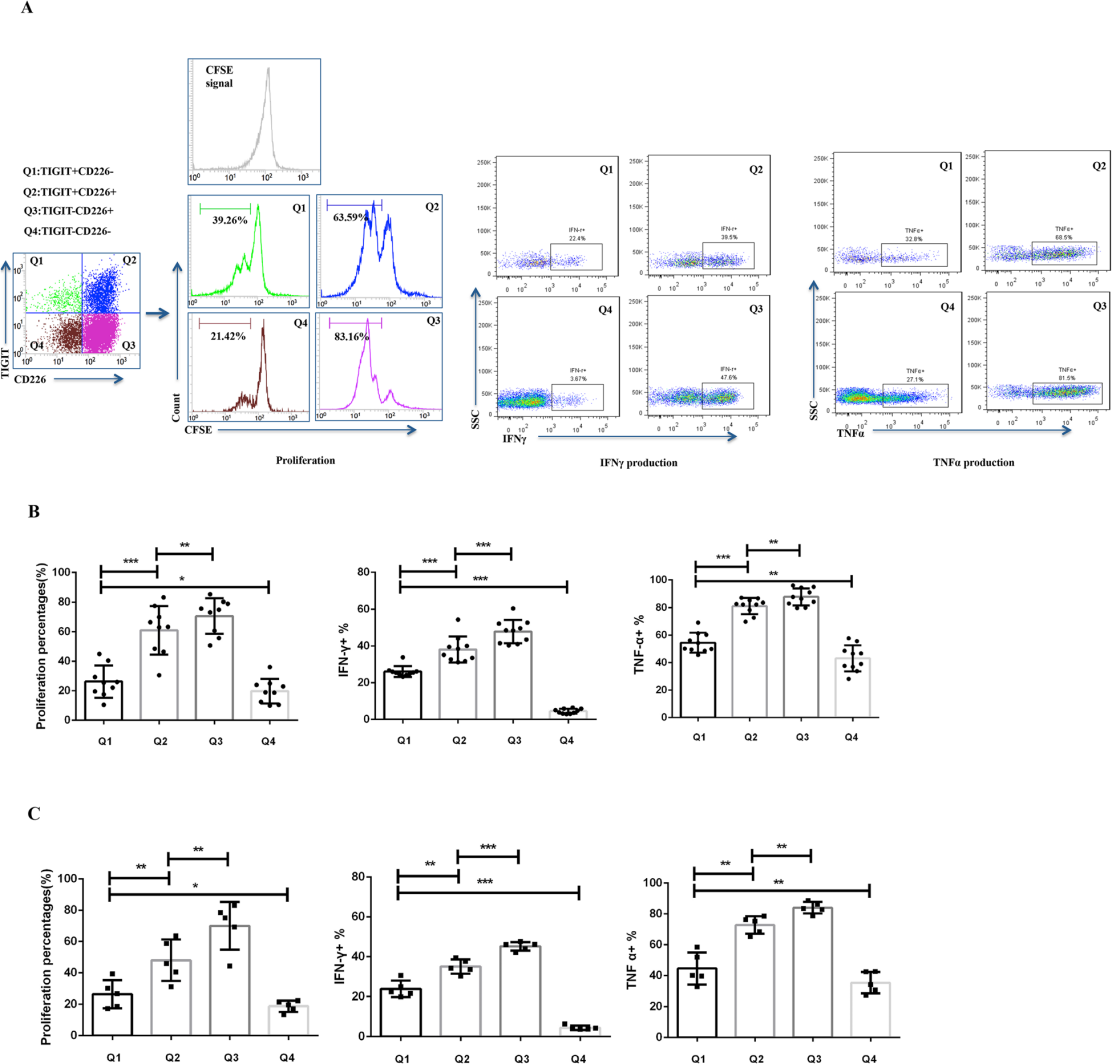
CD4 T cell subset function varied by TIGIT/CD226 phenotype. **a** Representative FACS plots showing the multi-parameter strategy for assessing the proliferation and cytokine production from CD4 T cells with different TIGIT/CD226 phenotypes. **b**, **c** Both in patients with DM (**b**) and HCs (**c**), the proliferation and cytokine production potential were the highest in the TIGIT–CD226+ subset, followed by in the TIGIT+CD226+, TIGIT+CD226−, and TIGIT−CD226− subsets. For the proliferation assay, 5 independent experiments evaluating a total of 9 patients with DM and 5 HCs were carried out; for intracellular cytokine staining, 5 independent experiments evaluating a total 10 patients with DM and 5 HCs were carried out. Data are shown as the mean ± SD, comparisons between DM and HCs were carried out using unpaired two-tailed *t* tests. *p < 0.05, **p < 0.01, ***p < 0.001

CFSE-based lymphocyte proliferation assay and intracellular IFN-γ and TNF-α staining were performed to determine the effector functions of CD4 T cells with different TIGIT/CD226 phenotypes (Fig. 2a). Both in patients with DM (*n* = 10) (Fig. 2b) and HCs (*n* = 5) (Fig. 2c), the percentage of proliferating cells was the highest in the TIGIT–CD226+ subset, followed by the TIGIT+CD226+, TIGIT+CD226−, and TIGIT−CD226− subsets. Additionally, the TIGIT−CD226+, TIGIT+CD226+, TIGIT+CD226−, and TIGIT−CD226− subsets showed the highest to lowest expression levels of IFN-γ and TNF-α. These results are consistent with the current understanding that CD226 is associated with proinflammatory Teff and TIGIT is an important negative regulator of CD4 T cell expansion and effector function [3, 6].

### TIGIT+CD226+ CD4 T cell effector function was enhanced in patients with DM

**Fig. 3:**
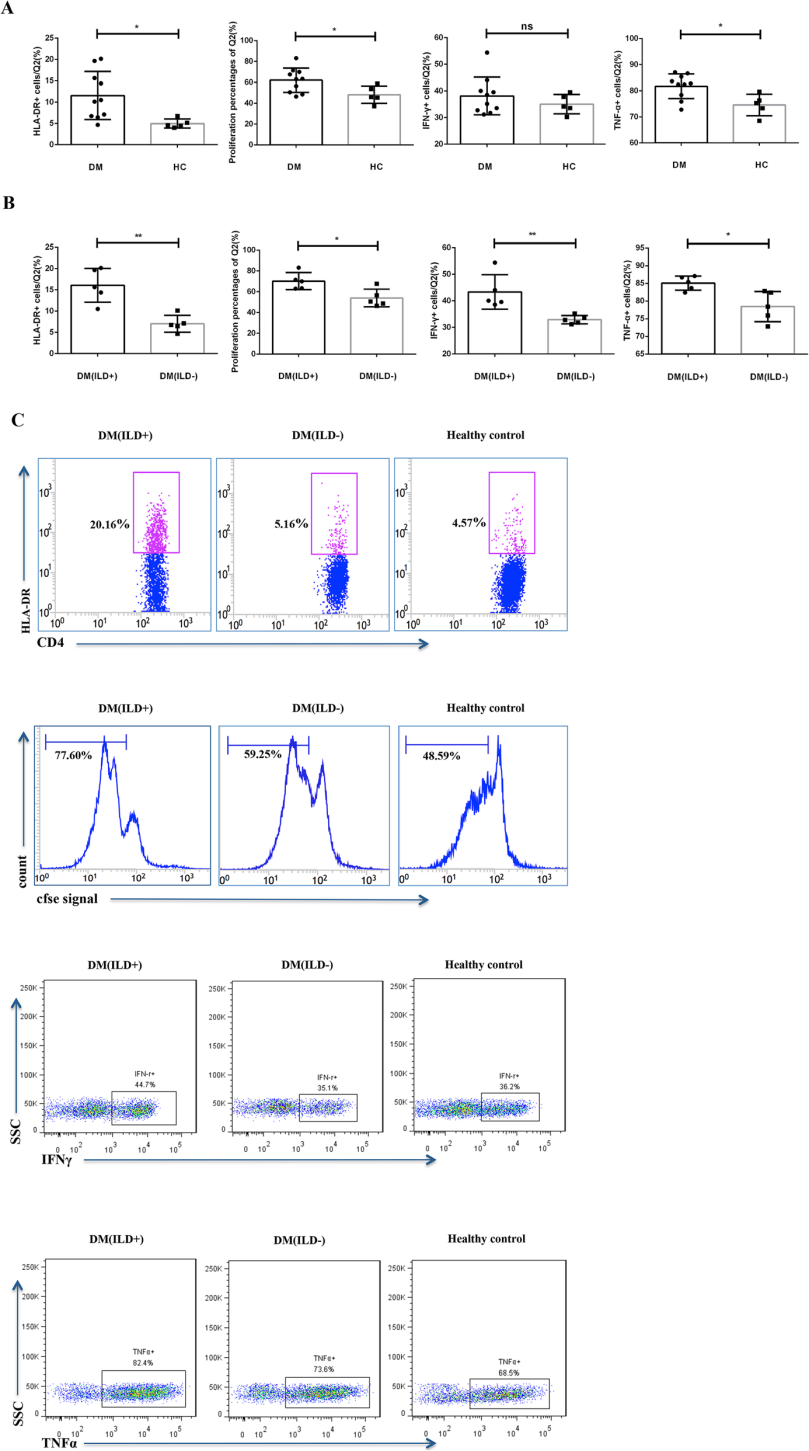
Effector function of TIGIT+CD226+ CD4 T cells was altered in patients with DM. **a** The functional comparisons between DM and healthy controls showed that expression of HLA-DR, the percentage of proliferating cells, and TNF-α production potential of TIGIT+CD226+ subset in DM were significantly higher than that of healthy controls. **b** In DM patients with ILD, the expression of HLA-DR, the percentage of proliferating cells, and cytokine production potential of TIGIT+CD226+ CD4 T cells were increased compared to in DM patients without ILD. **c** Representative FACS plots of different groups. Data are shown as the mean ± SD. Comparisons between two groups were carried out using unpaired two-tailed *t* tests. Bar graphs showed summary of 5 independent experiments with total 5 DM with ILD, 5 DM without ILD, and 5 HCs. Samples from all groups were included in each run. *p < 0.05, **p < 0.01, ***p < 0.001

The functional comparisons between DM and healthy controls showed that expression of HLA-DR, the percentage of proliferating cells, and TNF-α production potential of TIGIT+CD226+ subset in DM were significantly higher than that of healthy controls (Fig. 3a). Furthermore, the effector function of TIGIT+CD226+ CD4 T cells was correlated with the disease status: the HLA-DR expression on TIGIT+CD226+ CD4 T cells in DM patients with ILD (*n* = 5) was significantly higher than that in DM patients without ILD (*n* = 5) (Fig. 3b, 16.07 ± 3.990% vs 7.03 ± 1.976%, *p* = 0.0019). Similarly, in DM patients with ILD, the proliferation and cytokine production of TIGIT+CD226+ CD4 T cells was increased compared to in DM patients without ILD (Fig. 3b, proliferation percentages: 70.22 ± 8.198% vs 53.96 ± 8.488%, *p* = 0.0151; percentages of IFNγ+ cells/Q2: 43.36 ± 6.516% vs 32.92 ± 1.572%, *p* = 0.0083; percentages of TNF-α + cells/Q2 were 85.06 ± 1.993% and 78.46 ± 4.271%, *p* = 0.0140). Representative FACS plots of different groups are shown in Fig. 3c. These data suggest that TIGIT+CD226+ CD4 T cells are overactive in patients with DM, particularly in those with ILD.

### TIGIT+ CD4 T cells were generated from TIGIT–CD4 T cells following prolonged stimulation

**Fig. 4:**
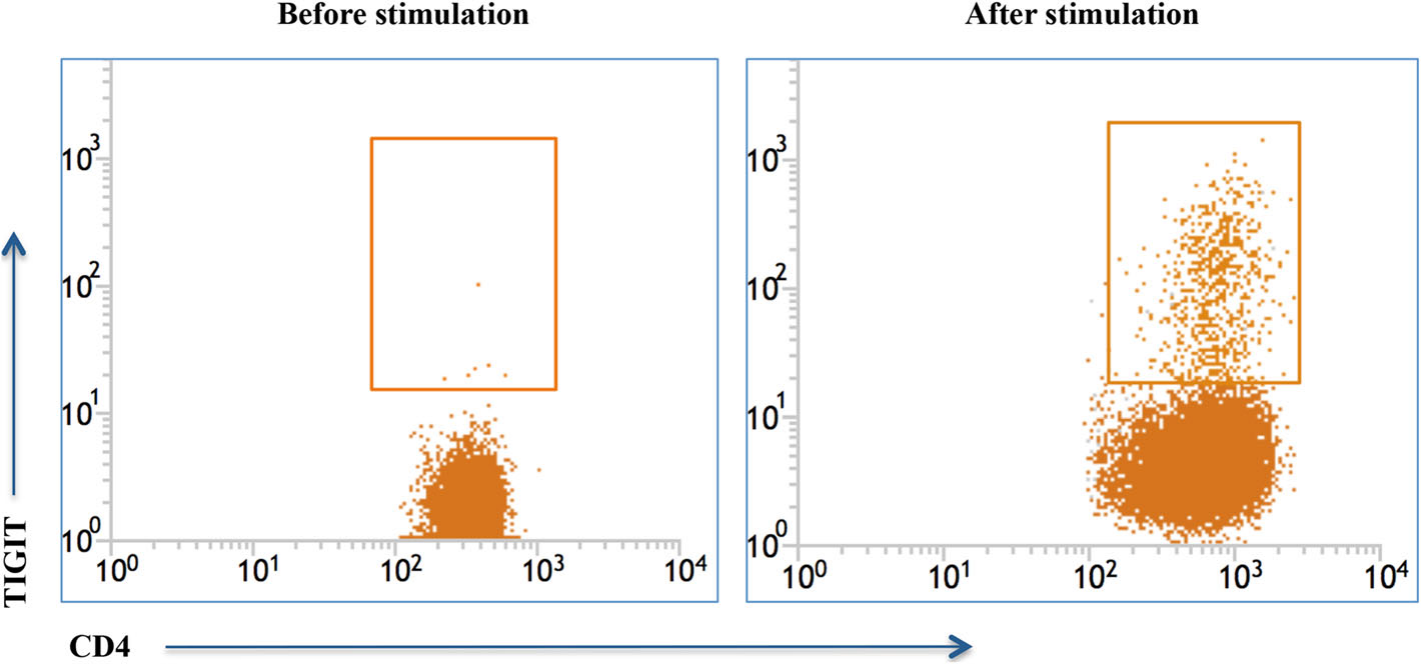
TIGIT expression in purified TIGIT-negative CD4 T cells after prolonged stimulation. Purified CD4+CD25−TIGIT− T cells were stimulated by anti-CD3/CD28 and were re-analyzed 72 h later. TIGIT-positive CD4 T cells were obviously generated from TIGIT-negative cells after 72-h stimulation. The graph was representative of 2 independent experiments with 3 DM and 1 HC

CD4+CD25–TIGIT– T cells were isolated from peripheral blood mononuclear cells from patients with DM and HCs by cell sorting on a BD FACSAria platform. Purified cells were stimulated with anti-CD3/CD28 and reanalyzed on a FACS JAZZ instrument at 72 h later. TIGIT+ CD4 T cells were clearly generated from CD4+CD25–TIGIT– T cells after 72 h of stimulation (Fig. 4).

### TIGIT+CD226+ CD4 T cells were suppressed significantly by anti-CD226 antibodies

**Fig. 5:**
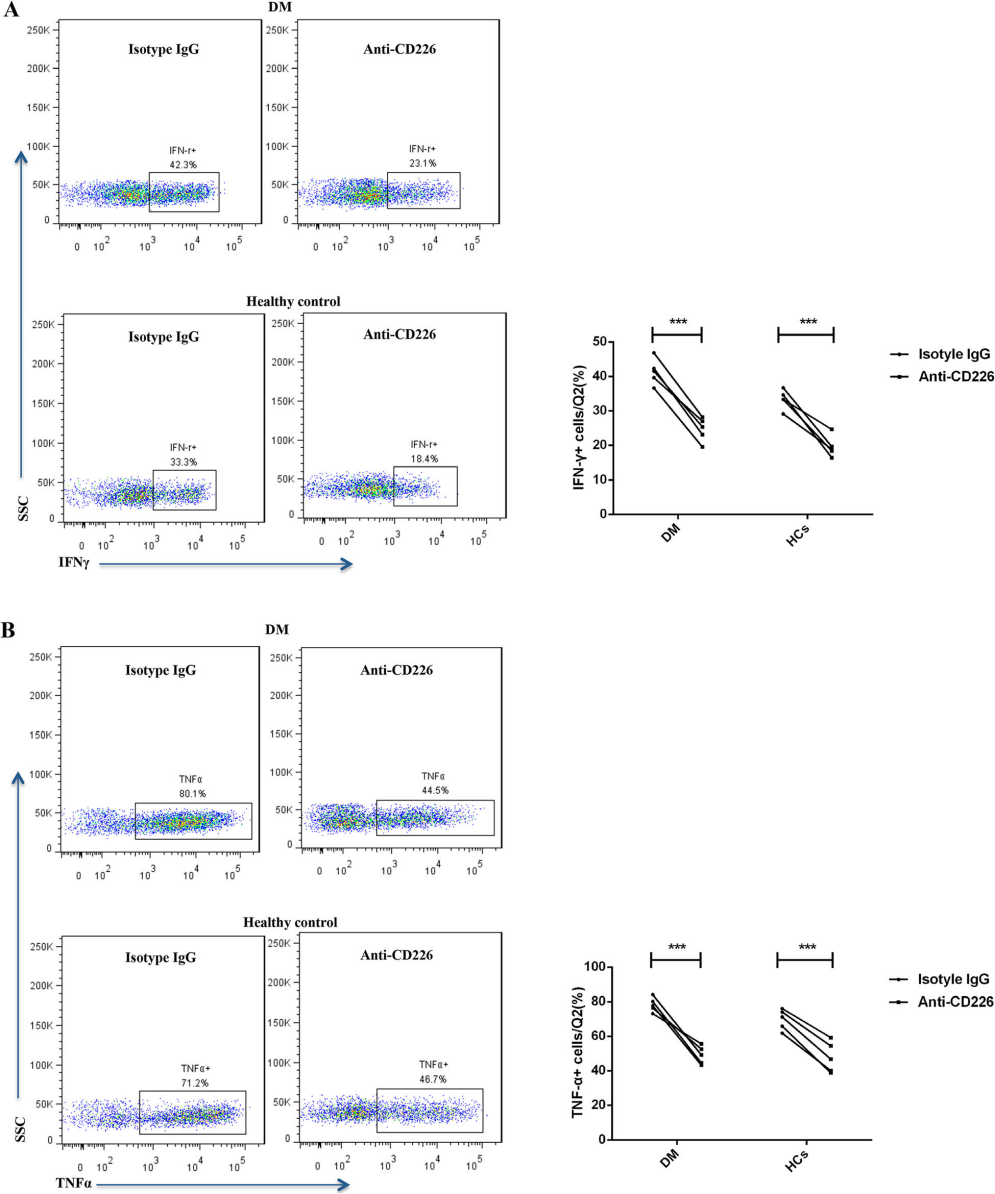
Anti-CD226 antibodies downregulated TIGIT+CD226+ CD4 T cell function. Representative FACS plots showing IFN-γ (**a**) and TNF-α (**b**) expression in TIGIT+CD226+ CD4 T cells after stimulation with or without anti-CD226 treatment (left panel). Graphs showing remarkably reduced production of IFN-γ (**a**) and TNF-α (**b**) from TIGIT+CD226+ CD4 T cells following CD226 blockade in both patients with DM and HCs (right panel). Comparisons between two groups were carried out using paired two-tailed *t* tests. Five independent experiments evaluating a total 5 patients with DM and 5 HCs were carried out. *p < 0.05, **p < 0.01, ***p < 0.001

To examine the potential for therapeutic intervention for the increased TIGIT+CD226+ CD4 T cell subset in patients with DM, we assessed the functional consequences of CD155, CD112, and anti-CD226 antibody treatments on TIGIT+CD226+ CD4 T cells. Compared to the isotype IgG-treated controls, CD226 blockade remarkably reduced IFN-γ and TNF-α production by TIGIT+CD226+ CD4 T cells in both patients with DM (*n* = 5) and HCs (*n* = 5) (Fig. 5). However, CD155 and CD112 only slightly decreased TNF-α production; this change was not significant.

## Discussion

In this study, for the first time, different T cells phenotypes based on co-expression of the immune checkpoint molecules TIGIT and CD226 were identified and characterized in patients with DM. Our data revealed that the percentages of TIGIT+CD226+ CD4 T cells were increased in patients with DM and that these percentages correlated positively with DM disease activity and closely related to lung involvement. Additionally, TIGIT+CD226+ CD4 T cells exhibited enhanced effector functions in patients with DM. Furthermore, this abnormal T cell subset was functionally suppressed by antibody blockade of CD226 in vitro.

The TIGIT/CD226 axis is a newly recognized pathway that regulates T cell function, with CD226 transmitting positive signals and TIGIT transmitting negative signals [3, 6]. Receptors TIGIT and CD226 not only compete with each other for their common ligands CD155 and CD112, but also interact with each other directly by disrupting receptor homodimerization [16]. Previous studies have investigated the individual roles of TIGIT and CD226 in several diseases [17–20]. However, the degree to which TIGIT and CD226 are co-expressed on T cells and their functional relationships have not been well-characterized. In this study, we first divided the T cells into four distinct subsets based on TIGIT/CD226 co-expression: TIGIT+CD226−, TIGIT+CD226+, TIGIT−CD226+, and TIGIT−CD226−. We then investigated the balances between these subpopulations and their association with DM disease activity. Six-color flow cytometry was performed to assess the cell surface expression of TIGIT and CD226 on T cells. As the functions of proinflammatory Teff and regulatory T cells differ [21], when the phenotypes of CD4 T cells were determined, we excluded regulatory T cells by gating the surface marker CD25.

We observed increased percentages of TIGIT+CD226+ CD4 T cells and profoundly decreased frequencies of TIGIT–CD226+ CD4 cells in patients with DM. There may be a shift from TIGIT–CD226+ CD4 cells to TIGIT+CD226+ CD4 cells under chronic stimulation, i.e., an altered balance between different TIGIT/CD226 phenotype subsets in patients with DM. Consistently, we detected TIGIT expression on purified TIGIT– CD4 T cells after chronic TCR-specific stimulation in vitro (Fig. 4). This alteration may result from a post-activation/refractory state [17, 22].

We show for the first time that TIGIT and CD226 expression profiles can be used to identify functionally distinct subsets of CD4 T cells. Notably, although TIGIT expression prevented T cell effector function to a certain extent (Fig. 2b, c, effector function of TIGIT+CD226+ subset was inferior to that of TIGIT–CD226+ subset), TIGIT+CD226+ T cells were still highly active (Fig. 2b,c, TIGIT+CD226+ subset showed much more robust effector function than the TIGIT−CD226− subset). Moreover, the effector function of TIGIT+CD226+ CD4 cells in patients with DM was abnormally enhanced compared with those in HCs (Fig. 3). These results improve the understanding of the positive association between increased TIGIT+CD226+ CD4 T cells and DM disease status. Although the TIGIT–CD226+ CD4 T subset possessed the most robust effector function because it was significantly reduced in DM, we did not focus on this subset in the present study.

ILD is one of the most common complications of DM and associated with worse outcomes and increased mortality [23]. Although the mechanisms underlying ILD in DM remain unclear, an increasing number of studies have suggested that the hyperactivity of T cells and a variety of inflammatory cytokines (IFN, TNF-α, TGF-β, etc.) play a critical role in the pathogenesis of ILD [24–26]. The elevated TIGIT+CD226+ CD4 subset with enhanced inflammatory cytokines production found in patients with DM complicated with ILD in the present study provided insight into the pathogenesis of DM-ILD.

To inhibit TIGIT+CD226+ CD4 T cells in vitro, recombinant CD155 and CD112 were first used to treat the cells to enhance the inhibitory function of TIGIT on T cells. However, our results indicated that CD155 and CD112 only slightly decreased cytokine production and the changes were not significant. Because CD155 can be induced after activation on both naïve and memory T cells [27], the ligand binding sites for the TIGIT receptor may already be close to saturation. Given that TIGIT and CD226 are co-expressed on the TIGIT+CD226+ cell subset and the CD226 antibody has been reported to effectively inhibit CD226 function [28–31], anti-CD226 antibodies were further used to suppress the function of TIGIT/CD226-expressing CD4 T cells. As expected, the CD226 antibody significantly decreased the stimulatory function of TIGIT+CD226+ T cells. Therefore, CD226 blockade is a promising approach for suppressing TIGIT+CD226+ CD4 T cell function in patients with DM. Our preliminary results suggest that CD226 blockade can inhibit abnormal TIGIT/CD226 double-positive CD4 T cells in DM. The influence of CD226 blockade on other CD226-expressing immune cells requires further investigation.

There were some limitations to the present study. First, our sample size was small. To eliminate the possible effects of drug therapy on TIGIT/CD226 expression, all peripheral blood samples were collected before treatment. Treatment-naïve patients with DM were difficult to recruit, limiting our sample numbers and preventing powerful stratification analyses. Further studies with larger cohort should be conducted on patients from different backgrounds and disease states (different MSAs subtypes, with and without cancer) to better elucidate the role of TIGIT+CD226+ CD4 T cell in DM. Second, a previous study showed that TIGIT and CD226 molecules can be used to delineate functionally distinct Treg subsets [32]; however, T regulatory cells were not evaluated in this study. Related investigations of T regulatory cells should be carried out. Third, the present study focused on the expression profile and function of TIGIT/CD226 expressing peripheral CD4 T cells of DM patients. However, to what degree TIGIT and CD226 are co-expressed on T cells at inflamed tissue sites (muscle, skin, and lung) is unclear. Further clarification of this issue will provide more insights into the role of this T cell subtype in the pathogenesis of DM.

## Conclusion

Our results support the opposing roles of TIGIT and CD226 in the regulation of T cell activity and show that the TIGIT and CD226 expression profiles can be used to identify functionally distinct subsets of CD4 T cells. We identified an elevated TIGIT+CD226+ subset with enhanced effector function in patients with DM and showed that TIGIT+CD226+ CD4 T cell activity can be suppressed by blocking CD226. These results suggest the therapeutic potential of targeting the TIGIT/CD226 axis.

## Data Availability

The datasets used and/or analyzed during the current study are available from the corresponding author on reasonable request.

## Abbreviations

TIGIT: T cell Ig and ITIM domain
DM: Dermatomyositis
HCs: Healthy controls
ILD: Interstitial lung disease
DLCO: Diffusing capacity of the lungs for carbon monoxide
APC: Allophycocyanin
PerCP: Peridin-chlorophyll protein
PE: Phycoerythrin
FITC: Fluorescein isothiocyanate
FACS: Fluorescent-activated cell sorting
IFN-γ: Interferon γ
TNF-α: Tumor necrosis factor α
CFSE: Carboxyfluorescein succinimidyl ester
MYOACT: Myositis Disease Activity Assessment
MSA: Myositis-specific antibody
